# A label-free fluorescent sensor for miR-21 based on sequentially coupled CHA–HCR dual-stage amplification and synergistic signal regulation by graphene oxide and SYBR Green I

**DOI:** 10.1039/d6ra08323d

**Published:** 2026-10-19

**Authors:** Can Qiao, Zhenliang Zhang, Yidan Sang, Mengyang Hu, Yafei Dong, Luhui Wang

**Affiliations:** a College of Life Science, Shaanxi Normal University Xi'an Shaanxi 710119 PR China dongyf@snnu.edu.cn; b College of Electrical and Information Engineering, Zhengzhou University of Light Industry Zhengzhou Henan 450000 PR China

## Abstract

MicroRNAs (miRNAs) are promising biomarkers for cancer diagnosis, and their sensitive and accurate detection is important for early cancer diagnosis. However, achieving sensitive miRNA detection and logic analysis while maintaining a simple workflow remains challenging. In this study, catalytic hairpin assembly (CHA) and hybridization chain reaction (HCR) were sequentially coupled to construct a dual-stage enzyme-free amplification circuit and integrated with a label-free fluorescence reporting strategy based on graphene oxide and SYBR Green I for logical recognition and sensitive detection of miR-21. CHA provides specific target recognition and primary signal amplification, and its product triggers the downstream HCR to generate dsDNA nanowires. The resulting dsDNA provides abundant SYBR Green I binding sites and enables continuous signal enhancement. Molecular simulations further supported the feasibility and kinetic behavior of the sequential cascade pathway. The modular cascade also produced a three-input AND logic response, demonstrating controllable logic operation. Under isothermal, enzyme-free, and label-free conditions, the sensor achieved a limit of detection of 184 pM and distinguished highly similar miRNA sequences at the single-base level. This CHA–HCR system provides a simple and reliable approach to miRNA analysis and shows potential for the convenient, sensitive detection of trace biomarkers.

## Abstract 2

Sequential CHA–HCR dual-stage amplification was integrated with GO/SYBR Green I signal regulation for label-free miR-21 detection, enabling low-background fluorescence output and a three-input AND logic response.

## Graphical abstract

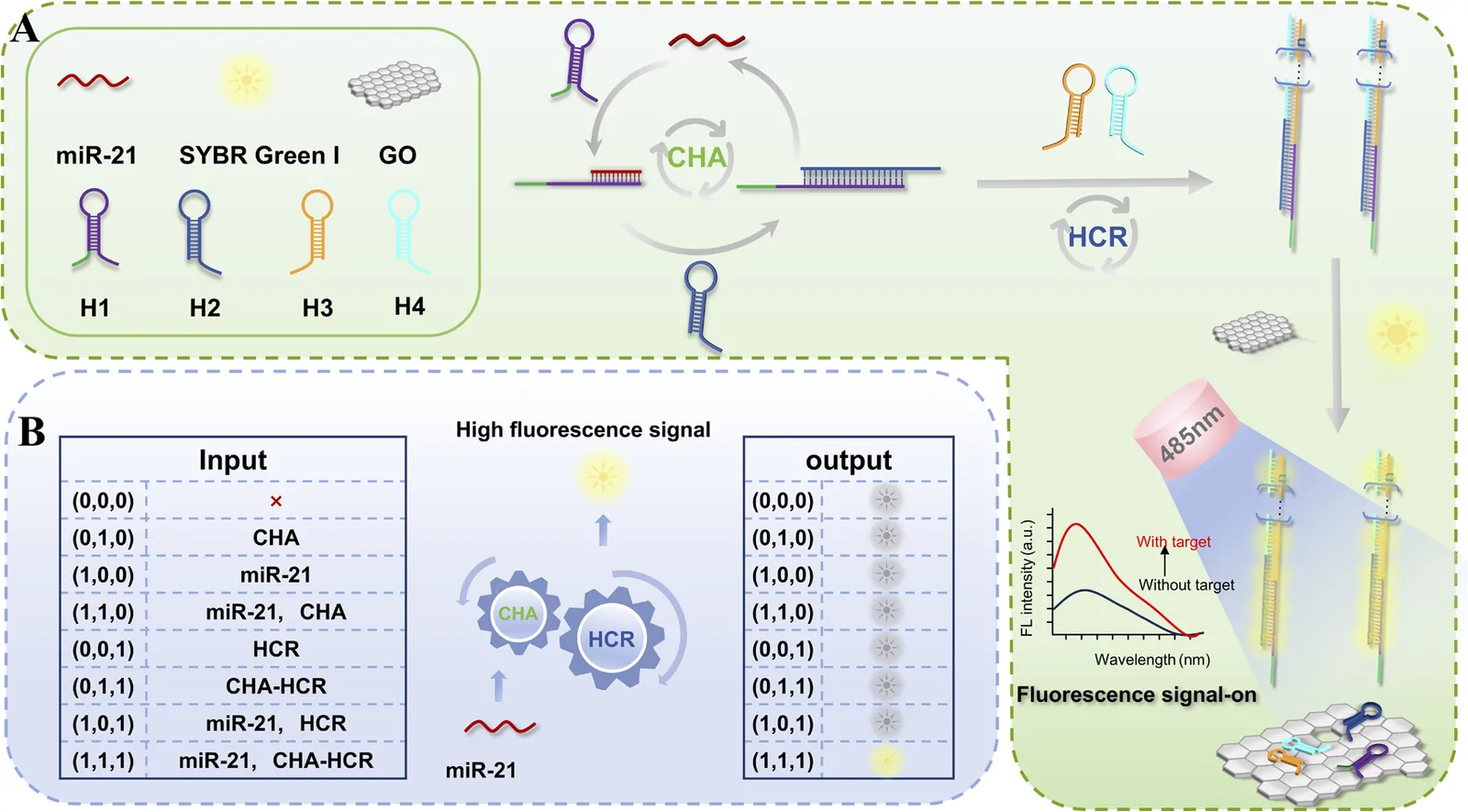

## Introduction

MicroRNAs (miRNAs) are a class of small non-coding RNA molecules with lengths of approximately 18–25 nucleotides.^1,2^ Extensive studies have demonstrated that abnormal miRNA expression is closely associated with tumor initiation and progression.^3,4^ Among them, the overexpression of miR-21 in serum has been reported in various malignant tumors, including lung cancer,^5^ prostate cancer,^6^ breast cancer,^7^ and pancreatic cancer,^8^ and miR-21 is widely recognized as an important cancer biomarker.^9^ However, the clinical application of miRNA analysis remains challenging due to their short length, low abundance, and high sequence similarity.^10,11^ Currently, conventional miRNA detection methods mainly include quantitative real-time PCR (qPCR),^12^ northern blotting,^13^ microarray analysis,^14^ and high-throughput sequencing. Although these techniques provide high sensitivity and specificity, they generally require complicated procedures and expensive instrumentation.^15^ Therefore, the development of simple, cost-effective, and highly sensitive miRNA detection strategies is highly desirable.

In recent years, various biosensing approaches have been developed for miRNA detection, including Raman spectroscopy,^16^ nanopore sensing,^17^ colorimetric assays,^18^ fluorescence spectroscopy,^19^ surface plasmon resonance,^20^ and electrochemiluminescence.^21^ Among these methods, strategies based on toehold-mediated strand displacement reactions (TMSDRs) have attracted increasing attention for miRNA analysis due to their operational simplicity, low cost, enzyme-free characteristics, and isothermal self-assembly capability.^22–24^ Catalytic hairpin assembly (CHA) and hybridization chain reaction (HCR)^25^ are two representative TMSDR-based amplification strategies, both of which are enzyme-free, efficient, and isothermal catalytic reactions. CHA achieves target-triggered cyclic amplification through nucleic acid hybridization and strand displacement,^26^ whereas HCR requires only a pair of hairpin probes containing complementary domains and generates long nicked dsDNA polymers upon target activation. Currently, CHA and HCR circuits have been widely applied in biosensing,^27^ bioimaging,^28,29^ nanorobotics,^30^ and molecular logic circuits.^31^ However, the amplification capability of individual DNA circuits remains limited. To overcome this limitation, cascade amplification strategies integrating CHA and HCR have been explored.^32^ For example, Li *et al.* developed a GO-AR system for live-cell imaging of circ-Foxo3.^33^ Similarly, Wang *et al.* developed a triple cascade amplification electrochemical biosensor for miRNA detection by integrating CHA, DNAzyme, and HCR modules.^34^ Therefore, integrating CHA and HCR into a cascade amplification system provides an effective strategy to achieve synergistic signal enhancement and improve analytical performance.^35^

Despite these advances, most reported amplification-based sensing strategies still rely on fluorophore- or quencher-labeled nucleic acid probes, which increase probe design complexity and assay cost.^36,37^ Alternatively, label-free sensing strategies based on DNA-binding dyes and functional nanomaterials have emerged as attractive approaches for simplified biosensing.^38,39^ SYBR Green I (SGI) is a widely used DNA-binding dye with high affinity toward the minor groove of double-stranded DNA (dsDNA).^40^ SGI exhibits weak fluorescence in the free or ssDNA-bound state but produces significantly enhanced fluorescence upon binding to dsDNA.^41^ In addition, graphene oxide (GO) can adsorb ssDNA through π–π stacking interactions, and its sp^2^-hybridized aromatic domains enable fluorescence quenching through fluorescence resonance energy transfer (FRET).^42^ Notably, GO and SGI exhibit complementary discrimination toward ssDNA and dsDNA, respectively.^41^ Their combination can effectively suppress background fluorescence, enhance target-dependent signals, and reduce nonspecific responses. Therefore, integrating GO and SGI as alternatives to conventional labeling strategies provides a promising approach for improving detection sensitivity while simplifying assay design.^43,44^

The proposed system sequentially couples catalytic hairpin assembly (CHA) with hybridization chain reaction (HCR), enabling specific miR-21 recognition and fluorescence signal amplification through upstream target recycling and downstream polymerization amplification. First, computer-assisted simulations were performed to verify the structural feasibility of the sequential triggering mechanism of the cascade circuit at the molecular level. Subsequently, an AND logic sensing system integrating target recognition, signal amplification, and logical response was constructed based on the synergistic interaction among cascade components, improving response controllability and reducing nonspecific outputs. Furthermore, GO-mediated background suppression and SGI-mediated dsDNA-responsive fluorescence generation were introduced to establish a label-free signal output strategy without fluorophore or quencher modification, providing a promising platform for sensitive and specific miR-21 detection.

## Materials and methods

### Reagents and instruments

The oligonucleotides and GoldORG fetal bovine serum (Uruguay origin) were supplied by Sangon Biotech (Shanghai) Co., Ltd. Graphene oxide was purchased from XFNANO Materials Technology Co., Ltd. SYBR Green I was obtained from Beijing Solarbio Science & Technology Co., Ltd. Tris–HCl, magnesium chloride (MgCl_2_), and sodium chloride (NaCl) were purchased from Xi'an Jingbo Biotechnology Co., Ltd.

### Computational simulation

Before the wet-lab experiments, NUPACK was used to predict the secondary structures of the hairpin probes and their interstrand hybridization. Visual DSD was then used to establish CHA, HCR, and CHA–HCR reaction models and to simulate strand displacement and cascade assembly.

### Construction of the CHA–HCR biosensor and fluorescence detection

Before use, hairpin probes H1, H2, H3, and H4 were separately heated at 95 °C for 5 min and slowly cooled to room temperature to form hairpin structures. The ssDNA sequences used in this study are listed in Table S1. Different concentrations of miR-21 were mixed with 50 nM H1 and 50 nM H2 in working buffer containing 20 mM Tris–HCl, 100 mM NaCl, and 5 mM MgCl_2_. The mixture was incubated in a water bath at 35 °C for 2 h for the CHA reaction. Subsequently, 100 nM H3 and 100 nM H4 were added, and the mixture was incubated for another 2 h to complete the HCR. Finally, 12.5 µL GO (25 µg mL^−1^) and 10 µL SGI (10×) were added, followed by incubation for 20 min. Fluorescence was measured using a BioTek SLXFA microplate reader with excitation and emission wavelengths of 485 and 528 nm, respectively. The fluorescence signal was monitored in real time.

### Construction of the cascaded AND logic circuit

The initial logic circuit contained 12.5 µL GO (25 µg mL^−1^) and 10 µL SGI (10×). Input 1 (2.5 µL miR-21), input 2 (2.5 µL H1/H2), and input 3 (5 µL H3/H4) were added in different combinations. Fluorescence was measured using a BioTek SLXFA microplate reader. An input state of “0” was represented by an equal volume of working buffer.

### Optimization and analytical performance

Initial reaction conditions were screened using a four-factor, three-level orthogonal design and were further refined by single-factor optimization. Sensitivity was evaluated from the fluorescence responses to different concentrations of miR-21. To simulate a complex sample matrix, spike-and-recovery experiments were performed in 10% fetal bovine serum containing 5, 20, or 50 nM miR-21. Analytical performance was assessed using recovery and relative standard deviation.

## Results and discussion

### Principle of the biosensing strategy

The principle of the sequential-coupled CHA–HCR system is shown in Scheme 1. The entire reaction is achieved through a target-triggered dual enzymeless amplification of CHA and HCR. As shown in Scheme 1A, the sensor primarily consists of four hairpin probes, with H1 and H2 participating in the upstream CHA reaction, and H3 and H4 used in the downstream HCR reaction. In the presence of miR-21, the target first binds to the toehold domain of H1 and opens the hairpin through toehold-mediated strand displacement reaction. The opened H1 subsequently hybridizes with H2 to form a stable H1–H2 complex. During this process, miR-21 is displaced and released. The released target can participate in subsequent CHA cycles, thereby generating multiple H1–H2 complexes and achieving primary signal amplification. The resulting H1–H2 complex then serves as the initiator of the downstream HCR. Specifically, the exposed toehold domain of the CHA product sequentially opens H3 and H4, leading to their alternating hybridization until the hairpin substrates are depleted. This process generates abundant DNA nanowires and provides secondary signal amplification. For fluorescence reporting (Scheme 1B), the DNA nanowires provide abundant binding sites for SYBR Green I (SGI), resulting in enhanced fluorescence. In the absence of miR-21, the unreacted hairpin probes and free SGI are adsorbed onto the graphene oxide (GO) surface, which effectively suppresses background fluorescence. Thus, the GO/SGI reporting system enables synergistic signal enhancement of amplification products and background suppression without requiring covalent modification of nucleic acid probes with fluorophores or quenchers. Scheme 1C illustrates the multi-input cascaded AND logic gate incorporated into the sensing system. The logic gate contains three input conditions that jointly determine the fluorescence output. Input 1 and input 2 activate the CHA module, and the resulting CHA product subsequently cooperates with input 3 to initiate the HCR module. Only when all three inputs are present, corresponding to the input state (1, 1, 1), is the complete sequential CHA–HCR cascade activated, producing a high fluorescence output. Overall, this system integrates multi-input cascade logic recognition with sequential CHA–HCR dual-stage enzyme-free signal amplification and low-background, label-free fluorescence reporting mediated by the combined action of graphene oxide and SYBR Green I, providing an enzyme-free and label-free strategy for miR-21 detection.

**Scheme 1:**
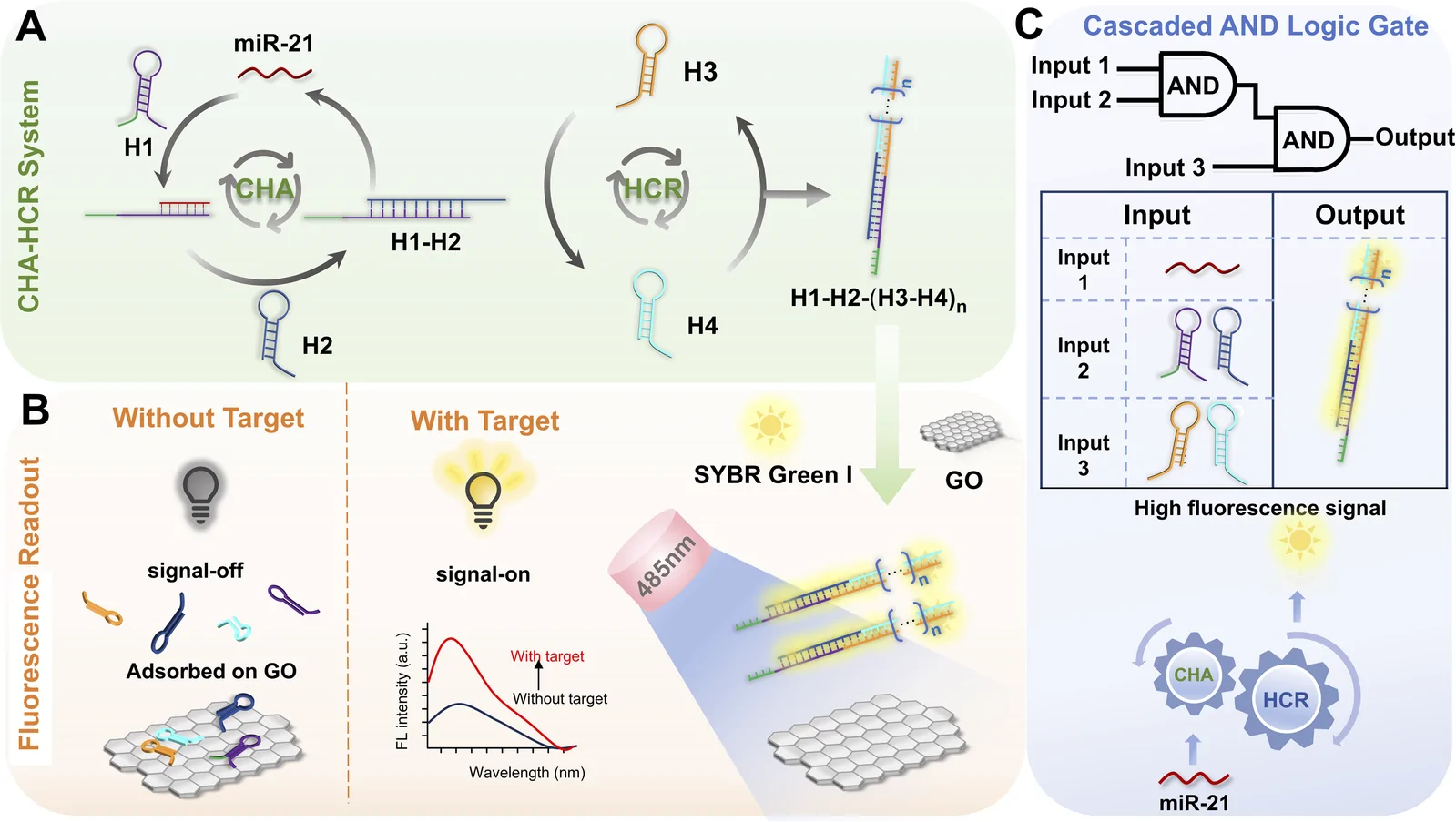
Schematic illustration of (A) sequentially coupled CHA–HCR dual-stage enzyme-free amplification, (B) graphene oxide/SYBR Green I-based label-free fluorescence reporting, and (C) the multi-input cascaded AND logic gate.

### Simulation analysis

Before the wet-lab experiments, NUPACK was used to evaluate the secondary structures of the hairpin probes and interstrand hybridization (Fig. S1). The probes formed the expected hairpin structures, and the target generated the designed intermediate complexes and long dsDNA products. Based on the sensing platform, an algorithmic framework with four functional modules for recognition, response, scanning, and decision-making was established (Table S2). Subsequently, the CHA, HCR, and integrated CHA–HCR reactions were simulated using Visual DSD (Fig. S2). In the complete cascade model, short DNA complexes were progressively extended into H1–H2–(H3–H4)_*n*_ long dsDNA products, supporting the sequential triggering of downstream HCR by the upstream CHA products. This process demonstrates continuous signal transmission from target recycling to polymeric chain extension. The kinetic simulation is shown in Fig. 1. Species sp4, sp7, and sp8 represent H1–H2–(H3–H4)_*n*_ dsDNA products formed at successive stages, whereas the other species are CHA–HCR intermediates. In the presence of the target, H1 and H2 were consumed during CHA. The resulting H1–H2 product subsequently drove H3/H4 assembly, and the concentration of long dsDNA increased until a plateau was reached. This behavior was consistent with substrate consumption and the gradual approach of the cascade to equilibrium. Overall, the simulation results support the feasibility of the sequential dual-stage CHA–HCR amplification design and provide a theoretical basis for the subsequent experiments.

**Fig. 1:**
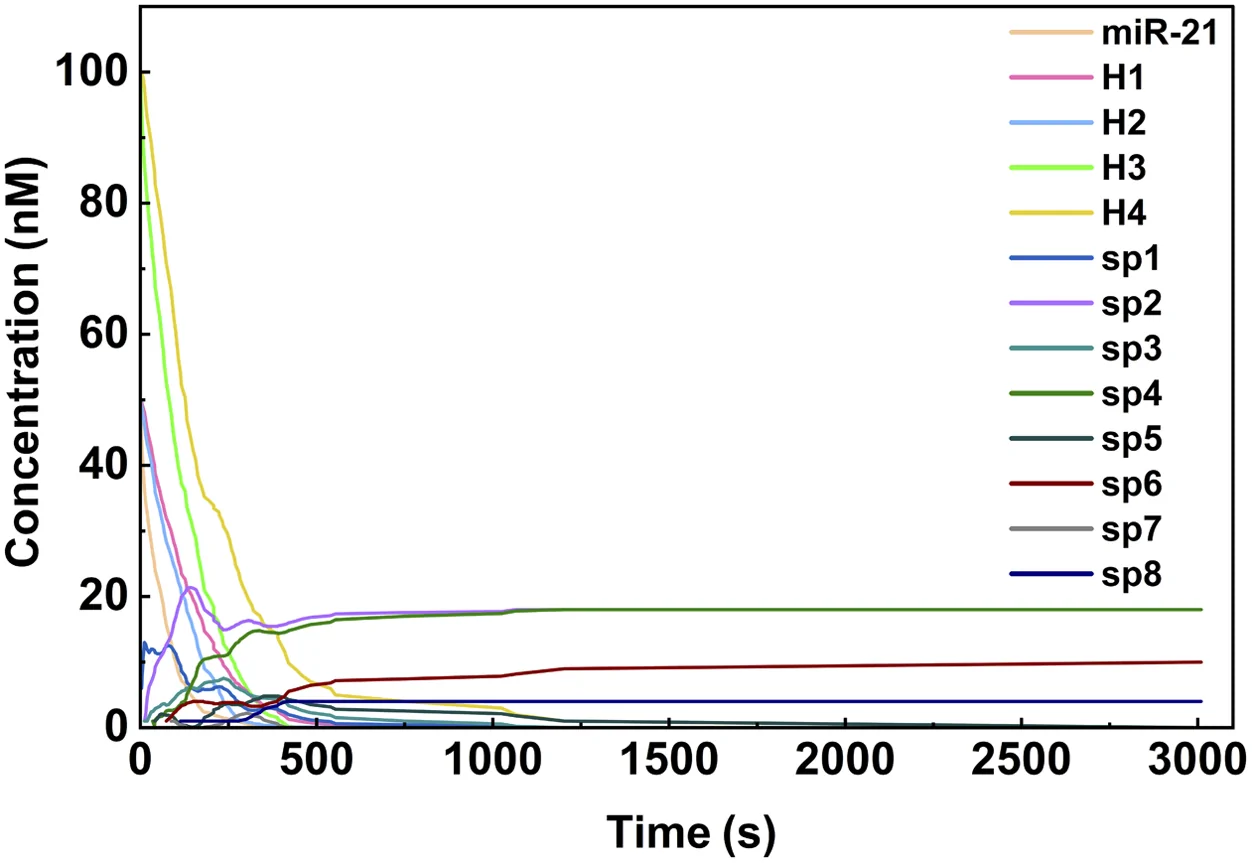
Dynamic concentration profiles of the nucleic acid species in the CHA–HCR system simulated using Visual DSD.

### PAGE verification

To further validate the sensing principle, 12% native polyacrylamide gel electrophoresis (PAGE) was performed to verify the feasibility of miR-21-triggered CHA and CHA–HCR cascade amplification. As shown in Fig. 2A, a new band with slightly slower migration than free H1 appeared in lane 4, indicating that miR-21 hybridized with H1 and opened its hairpin structure. Only the individual H1 and H2 bands were observed in lane 5, with no detectable product band. In contrast, lane 6 exhibited a new band with slower electrophoretic mobility and higher molecular weight, confirming that miR-21 effectively triggered the CHA reaction between H1 and H2 to form the H1–H2 dsDNA product.

**Fig. 2:**
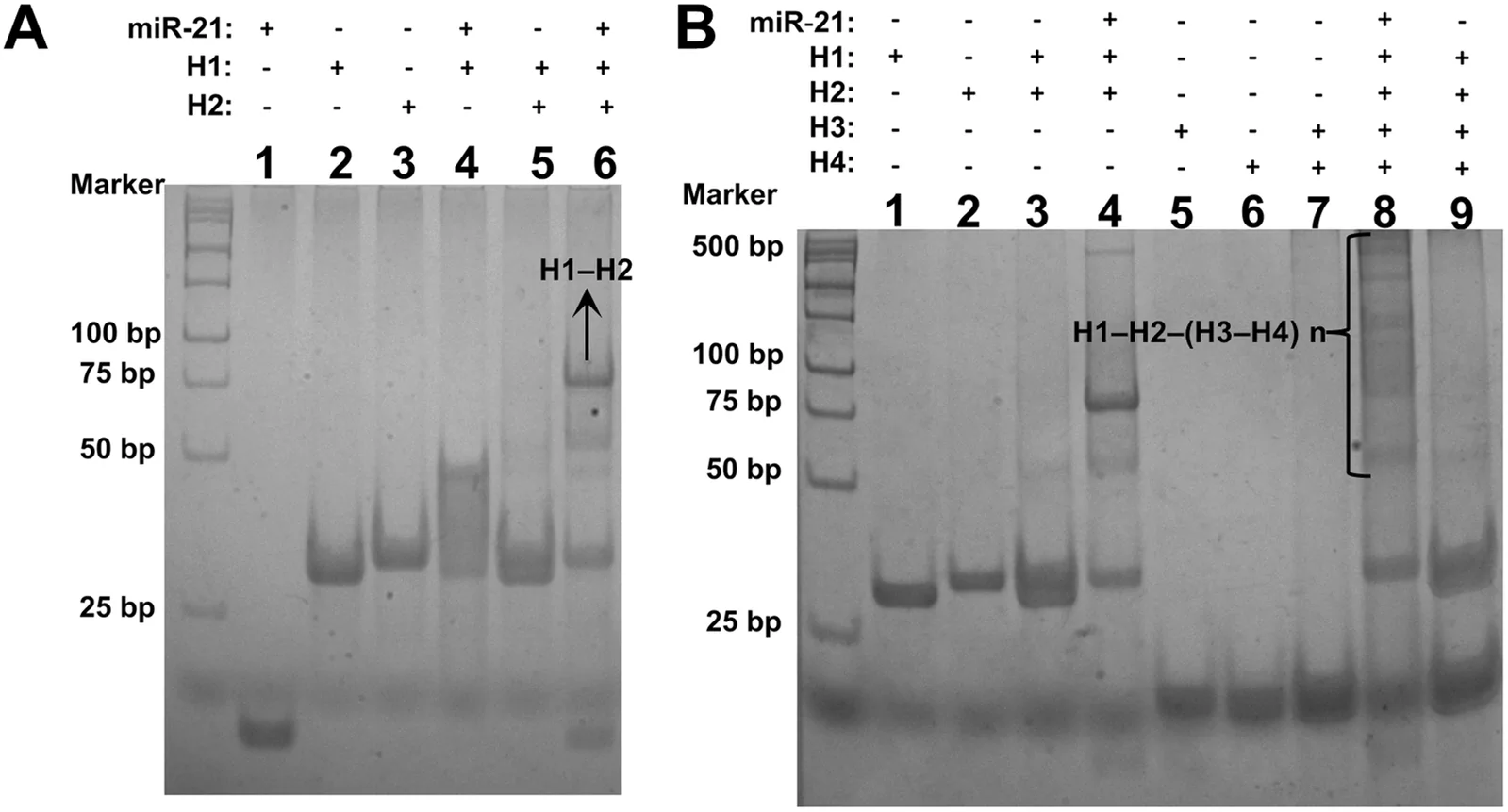
PAGE analysis of miR-21-triggered CHA amplification (A) and CHA–HCR dual-stage cascade amplification (B). (A) Lane 1, miR-21; lane 2, H1; lane 3, H2; lane 4, miR-21 + H1; lane 5, H1 + H2; lane 6, miR-21 + H1 + H2. (B) Lane 1, H1; lane 2, H2; lane 3, H1 + H2; lane 4, miR-21 + H1 + H2; lane 5, H3; lane 6, H4; lane 7, H3 + H4; lane 8, miR-21 + H1 + H2 + H3 + H4; lane 9, H1 + H2 + H3 + H4. The H1–H2 duplex and H1–H2–(H3–H4)_*n*_ polymer products are indicated by an arrow and a bracket, respectively. The corresponding uncropped gel images are provided in Fig. S4 and S5 in the SI.

The complete CHA–HCR cascade was further examined by PAGE (Fig. 2B). In the absence of miR-21, the four hairpin probes remained stable and showed no obvious spontaneous assembly (lane 9). After miR-21 was added (lane 8), the individual probe bands were replaced by ladder-like bands with low mobility near the top of the gel. These bands indicated the formation of high-molecular-weight DNA polymers generated by HCR, supporting target-triggered activation of the complete CHA–HCR cascade.

### Fluorescence feasibility verification

Fluorescence measurements were performed to further evaluate the feasibility of the sensing strategy (Fig. 3A). Both the miR-21/H1 and H1/H2 systems showed low fluorescence. After miR-21 initiated CHA, the resulting H1–H2 duplexes provided binding sites for SYBR Green I (SGI), resulting in enhanced fluorescence. The subsequent addition of H3 and H4 triggered downstream HCR and generated long dsDNA products with abundant SGI-binding sites, leading to further signal amplification. In contrast, the four-hairpin system without miR-21 remained stable and produced only low background fluorescence.

**Fig. 3:**
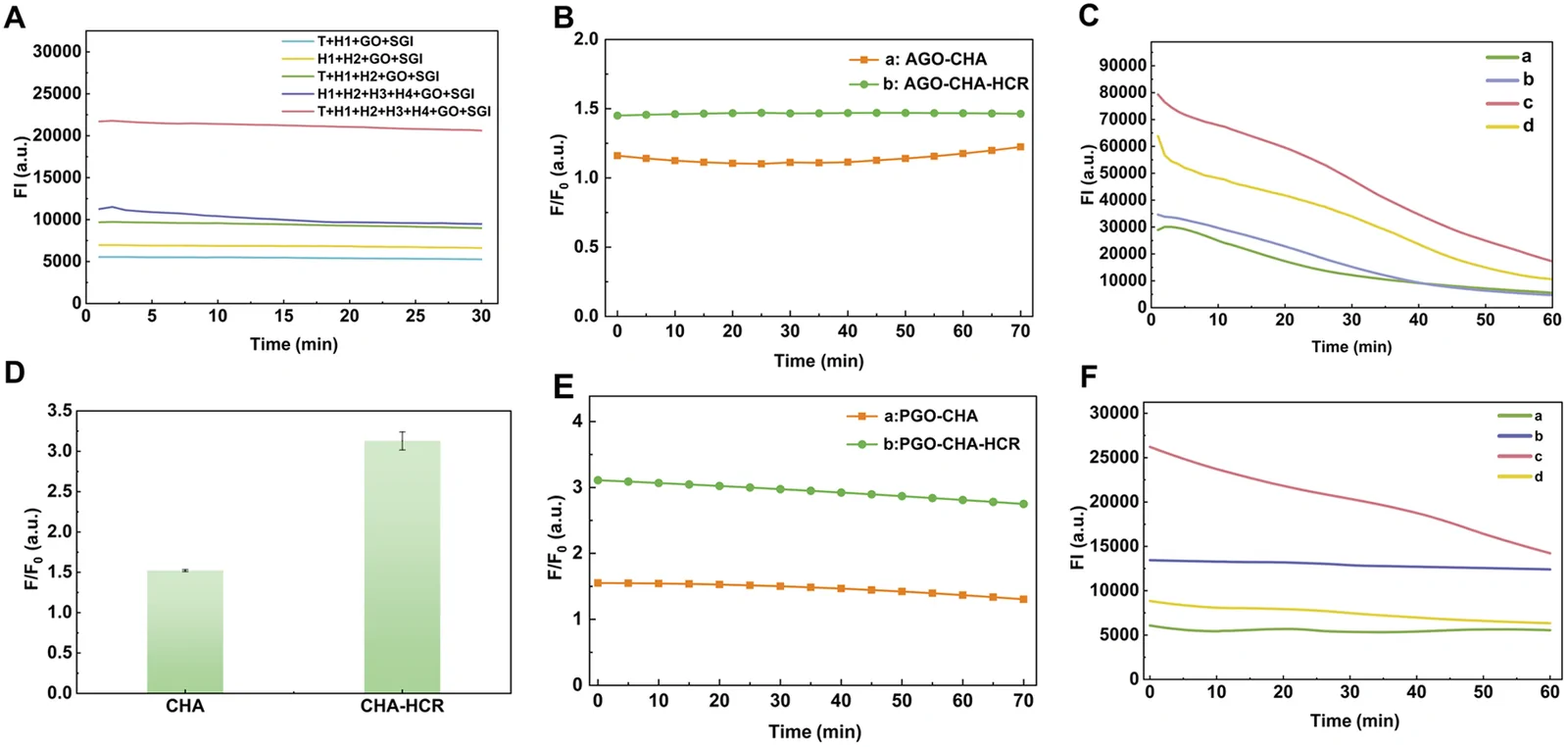
Fluorescence responses of SYBR Green I under different reaction conditions. (A) Fluorescence feasibility of the CHA and CHA–HCR systems in the absence of graphene oxide (GO). (B) Fluorescence ratios of the CHA and CHA–HCR systems without GO. (C) Fluorescence responses under different reaction conditions without GO: (a) H1 + H2; (b) miR-21 + H1 + H2; (c) miR-21 + H1 + H2 + H3 + H4; (d) H1 + H2 + H3 + H4. (D) Fluorescence intensities of CHA and CHA–HCR amplification in the presence of GO. (E) Fluorescence ratios of the CHA and CHA–HCR systems with GO. (F) Fluorescence responses under different reaction conditions with GO: (a) H1 + H2; (b) miR-21 + H1 + H2; (c) miR-21 + H1 + H2 + H3 + H4; (d) H1 + H2 + H3 + H4.

To evaluate the role of GO in the sensing system, reactions without GO (AGO) and with GO (PGO) were compared for both CHA and CHA–HCR amplification. In the AGO system (Fig. 3B and C), free hairpin probes readily bound SGI, resulting in high background fluorescence and a low fluorescence ratio. In the PGO system (Fig. 3D–F), GO effectively suppressed background fluorescence through its quenching effect, resulting in a significantly improved fluorescence response compared with the AGO system. Moreover, the fluorescence signal generated by CHA–HCR cascade amplification was markedly higher than that produced by CHA alone. After basal background correction, the no-target system showed a relative background contribution of 31.6%, while the target-activated system retained a 3.17-fold fluorescence response (Table S3). These results demonstrate that GO effectively suppresses background fluorescence, while the sequential coupling of CHA and HCR further enhances fluorescence output.

### Logic operation of the sensing platform

A three-input AND logic gate was established to assess the logic response of the CHA–HCR platform. miR-21, H1/H2, and H3/H4 were defined as input 1, input 2, and input 3, respectively (Fig. 4A), and fluorescence served as the output. Based on the responses of the different input combinations, 20 000 a.u. was set as the logic threshold. Fluorescence below this value was defined as output “0”, whereas fluorescence above this value was defined as output “1”. The corresponding truth table is shown in Fig. 4B.

**Fig. 4:**
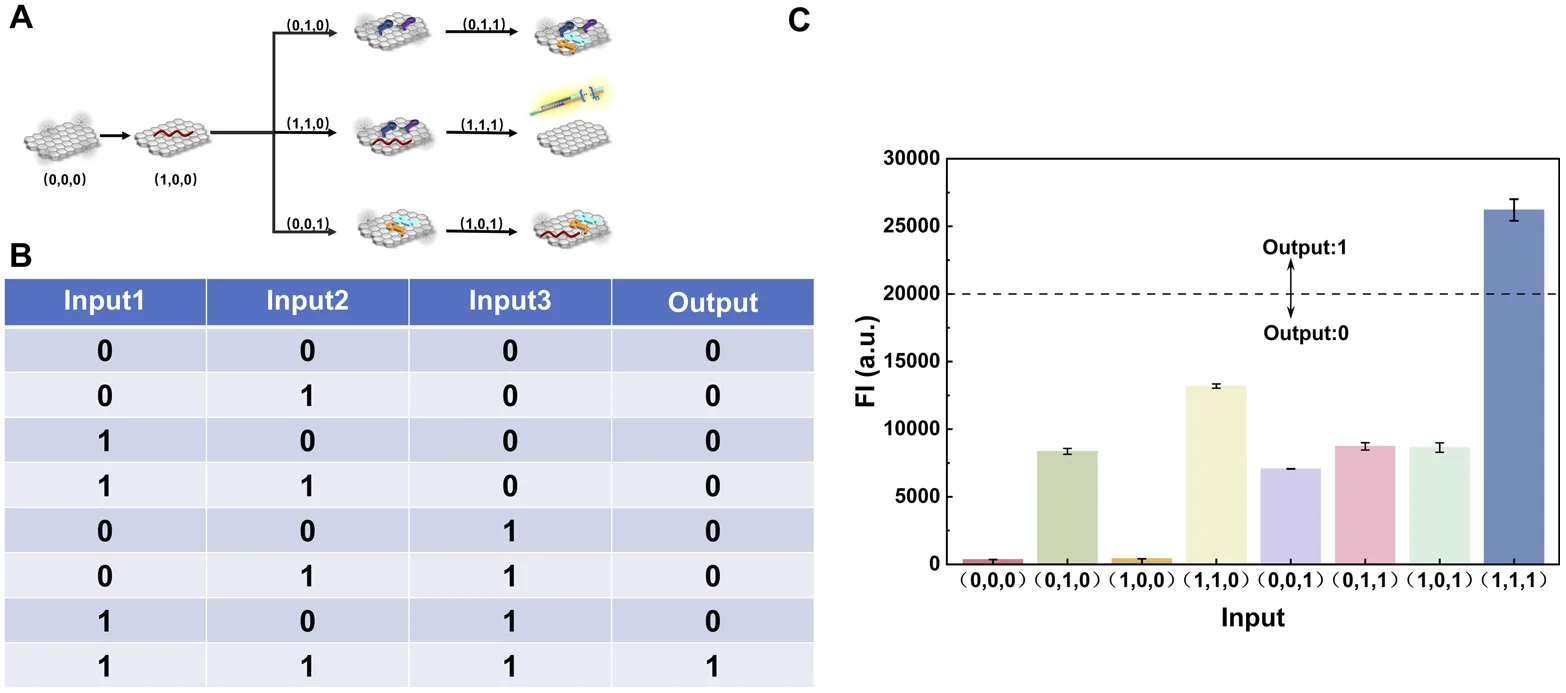
Design principle (A), truth table (B), and fluorescence validation (C) of the cascaded AND logic gate.

The fluorescence responses to different input combinations are shown in Fig. 4C. Except for the (1, 1, 1) state, all other input combinations generated fluorescence signals below the threshold. The absence of any component required to initiate or sustain the complete CHA–HCR cascade prevented sufficient formation of dsDNA products, resulting in an output of “0”. Only when all three inputs were present was the complete CHA–HCR cascade activated, producing a fluorescence signal above the threshold and an output of “1”. The experimental results were consistent with the truth table, confirming the three-input AND logic response of the platform. This Boolean response illustrates the modular organization of the platform and allows stepwise control of the sequential CHA and HCR modules.

### Orthogonal design

To improve the sensitivity of miR-21 detection, a four-factor, three-level orthogonal experiment was conducted to preliminarily screen the key parameters of the reaction system. The experimental design and results are presented in Tables S4 and S5. The optimal fluorescence ratio was obtained under the conditions of group 1 (*A*_1_*B*_1_*C*_1_*D*_1_), corresponding to an HCR reaction time of 120 min, an H1/H2 ratio of 0.75 : 1, a GO concentration of 10 µg mL^−1^, and an SYBR Green I concentration of 1×. These parameters were therefore used as the initial conditions for subsequent optimization.

Further analysis of the mean response (*K*_*ji*_) and range (*R*_*ji*_) for each factor showed that GO concentration had the largest effect on the fluorescence response. GO concentration was therefore optimized first, followed by the other reaction parameters.

### Optimization of reaction conditions

Based on the orthogonal experiment, GO concentration, GO incubation time, SYBR Green I concentration, reaction temperature, and reaction time were further optimized individually. As shown in Fig. 5A, a GO concentration of 25 µg mL^−1^ minimized the effect of background fluorescence on the sensing system, indicating effective background suppression without excessive attenuation of the target response. The fluorescence signal stabilized after 20 min of GO incubation, suggesting that adsorption between GO and the nucleic acid components had approached equilibrium (Fig. S3). Therefore, an incubation time of 20 min was selected for subsequent experiments. An SYBR Green I concentration of 1× produced the highest fluorescence ratio (Fig. 5B), providing sufficient staining of the dsDNA products while avoiding the high background associated with excess dye. The effect of reaction temperature was subsequently investigated (Fig. 5C), and the optimal response was obtained at 35 °C. Further increases in temperature may destabilize nucleic acid structures or disturb reaction kinetics, thereby reducing reaction efficiency. Thus, 35 °C was selected as the optimal reaction temperature. As the cascade assembly proceeded, the fluorescence signal increased with reaction time and approached a plateau after 120 min (Fig. 5D), indicating that the formation of long dsDNA products was close to saturation. Accordingly, 120 min was selected as the optimal reaction time to maximize signal gain while avoiding unnecessary reaction time and potential nonspecific interference.

**Fig. 5:**
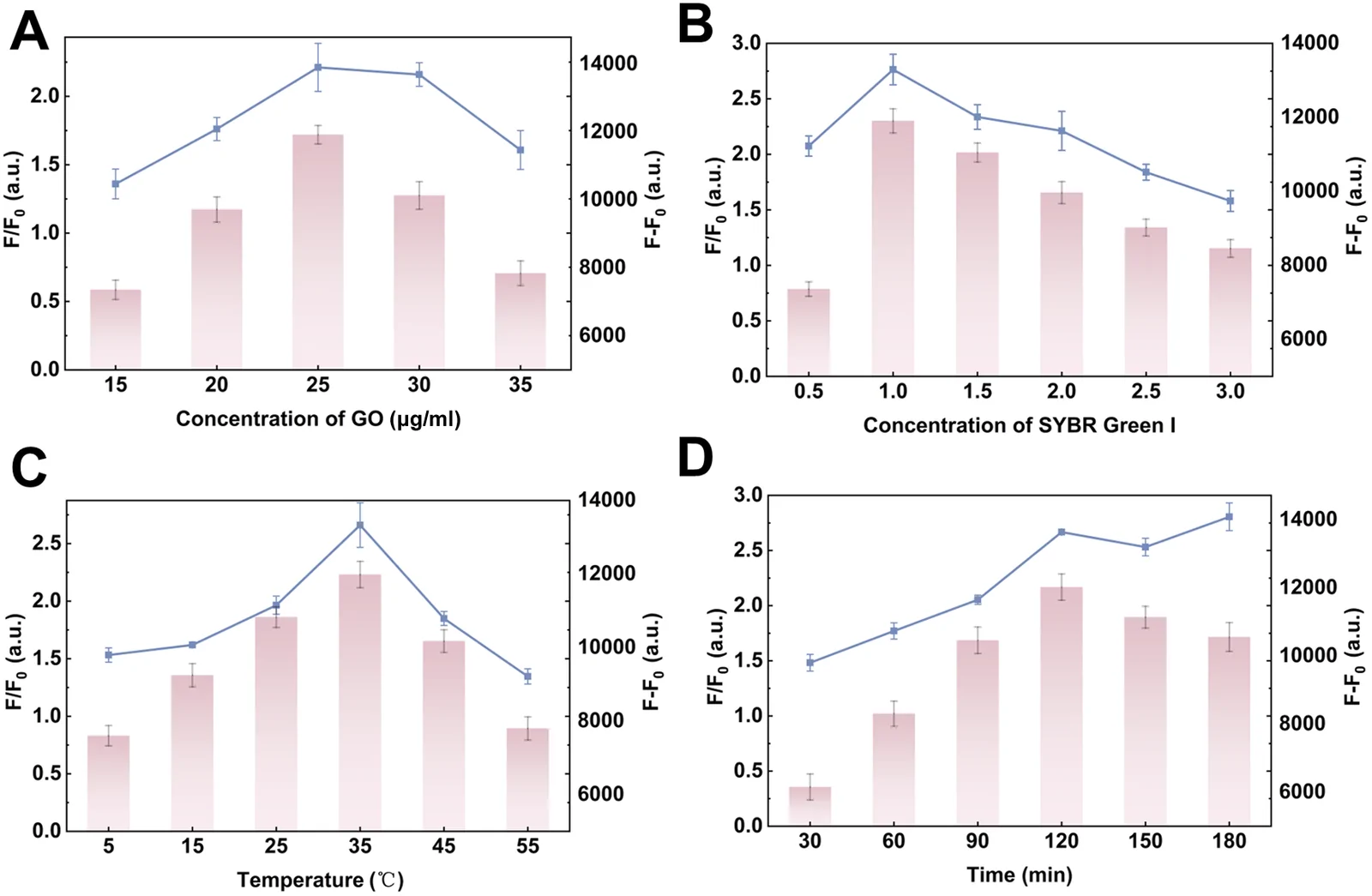
Optimization of GO concentration (A), SYBR Green I concentration (B), reaction temperature (C), and HCR time (D). *F* − *F*_0_ represents the fluorescence intensity difference, and *F*/*F*_0_ represents the fluorescence intensity ratio.

### Sensitivity

The analytical performance of the sensor was evaluated under the optimized conditions. Different concentrations of target miR-21 were introduced to investigate the fluorescence response of the biosensor. The fluorescence intensity increased with the miR-21 concentration (Fig. 6A), and a good linear relationship was obtained over the range of 0.2–70 nM (Fig. 6B). The calibration equation was *y* = 8113.24419 + 357.78314*x* (*R*^2^ = 0.99798), where *y* represents the fluorescence intensity and *x* represents the miR-21 concentration (nM). Based on LOD = 3*σ*/*S*, the limit of detection was calculated to be 184 pM.

**Fig. 6:**
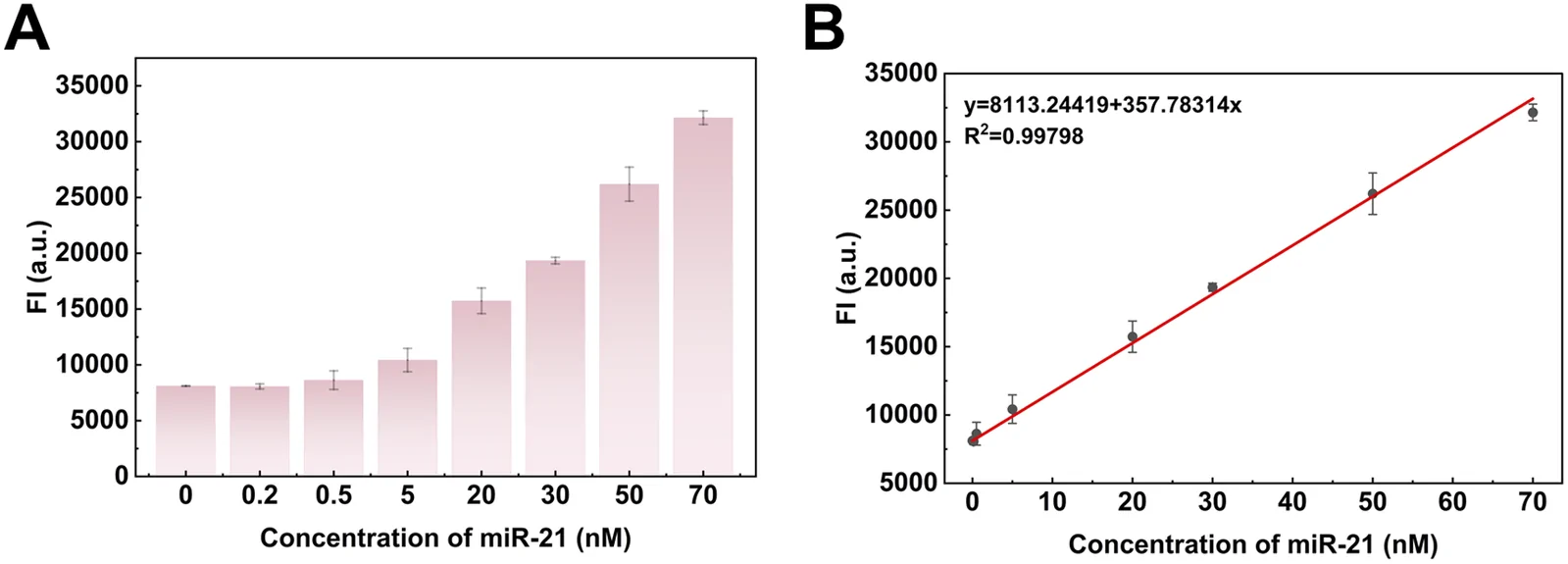
Fluorescence responses to different miR-21 concentrations under the optimized conditions (A) linear calibration curve of fluorescence intensity *versus* miR-21 concentration (B).

### Specificity

Due to the high sequence similarity among miRNA family members, interference from other RNAs may occur in complex samples. Therefore, under the optimized conditions, single-base mismatch (SM), double-base mismatch (DM), triple-base mismatch (TM), miR-155, miR-141, let-7a, miR-133a, and miR-128 at equal concentrations were used as interfering sequences and compared with the target miR-21 to evaluate the specificity of the sensor. As shown in Fig. 7, miR-21 produced a high fluorescence signal. The SM and DM signals were clearly lower than that of miR-21, whereas the TM and non-target miRNA signals were close to the blank. These results demonstrate that the sensing system can effectively discriminate miR-21 from mismatched sequences and other non-target miRNAs, indicating good specificity for miR-21 recognition.

**Fig. 7:**
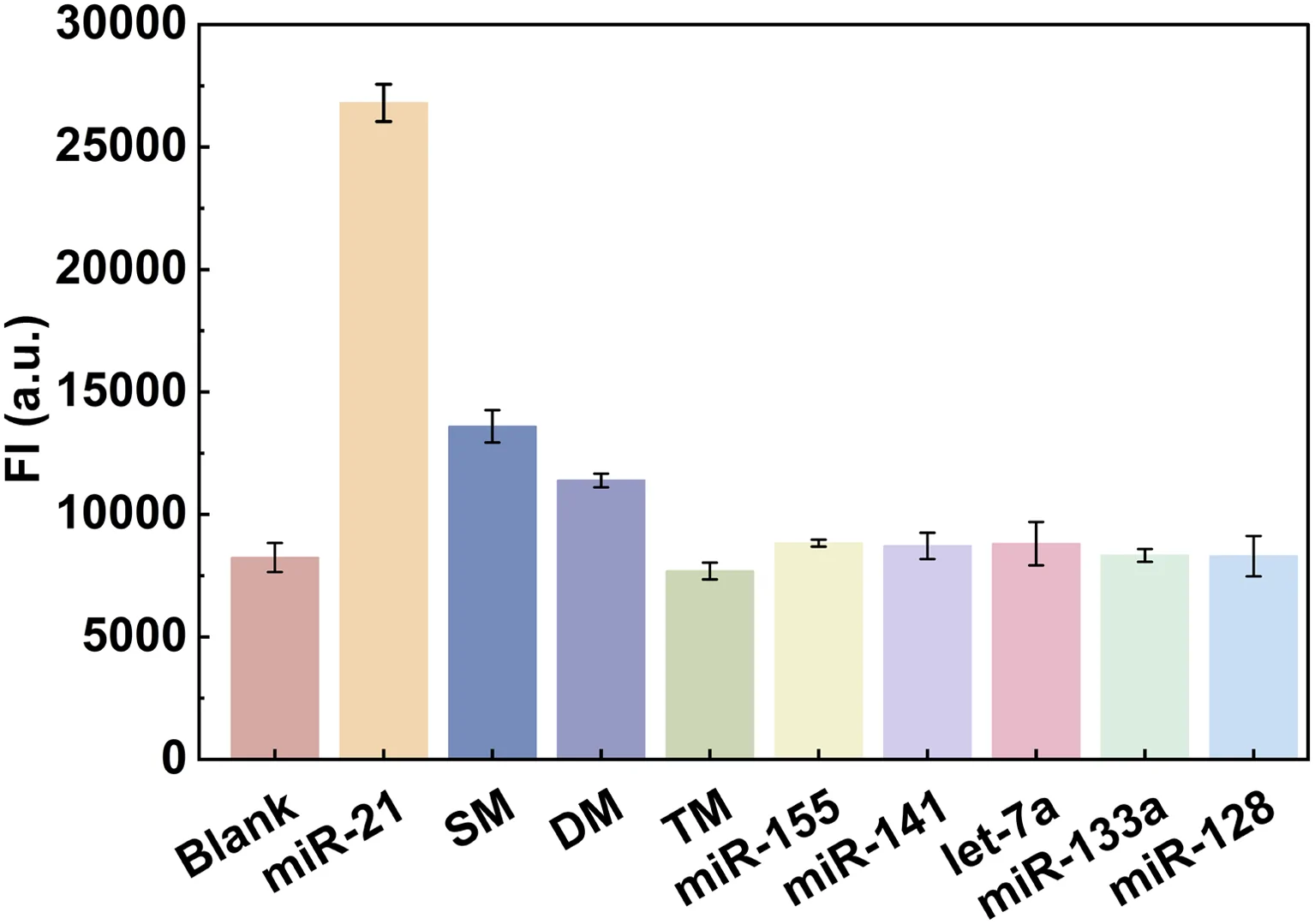
Specificity evaluation of the CHA–HCR sensing system for miR-21 detection.

### Real sample analysis and comparison with other methods

Complex bioactive components in serum may interfere with the detection process. To further evaluate the analytical performance of the proposed biosensor in complex biological matrices, spike-and-recovery experiments were performed in 10% fetal bovine serum (FBS) containing miR-21 at concentrations of 5, 20, and 50 nM. The recoveries ranged from 99.0% to 103.2%, with relative standard deviations (RSDs) of 1.51–4.33% (Table 1). These results indicate that the proposed biosensor exhibits good accuracy and repeatability in the 10% fetal bovine serum matrix, providing a basis for its further application to the analysis of complex biological samples. Compared with the reported miR-21 detection methods (Table S6), the proposed method offers a competitive detection limit and is free of enzymes and fluorescent labels, demonstrating that the CHA–HCR cascade strategy is a simple and sensitive detection platform.

**Table 1:** Analysis of miR-21 spiked into 10% fetal bovine serum

Added (nM)	Found (nM)	Recovery (%)	RSD (%)
5	4.95	99	1.86
20	20.64	103.2	1.51
50	51.31	102.62	4.33

## Conclusions

In conclusion, an enzyme-free and label-free fluorescent sensing platform for miR-21 detection was developed by integrating sequentially coupled CHA–HCR cascade amplification with fluorescence reporting based on graphene oxide and SYBR Green I. The CHA–HCR circuit enables target recycling and continuous signal amplification, while the combined action of graphene oxide and SYBR Green I provides low-background fluorescence output through background suppression and dsDNA-responsive signal enhancement. The proposed platform achieved sensitive and selective miR-21 detection with a detection limit of 184 pM and showed reliable analytical performance in a 10% fetal bovine serum matrix. In addition, the sensing strategy avoids covalent modification of nucleic acid probes with fluorophores or quenchers and does not require protein enzymes, thereby simplifying probe design and the overall assay procedure. The integration of sequential cascade amplification with molecular logic operation further provides both signal amplification and logical discrimination functions, offering a useful approach for expanding the functionality of enzyme-free nucleic acid sensing systems. Overall, the work combines dual-stage enzyme-free amplification with label-free fluorescence reporting, providing a simple and reliable analytical strategy for miRNA detection.

## Conflicts of interest

There are no conflicts to declare.

## Supplementary Material

RA-016-D6RA08323D-s001

## Data Availability

All experimental data generated and analyzed in this study are included within the article and its supplementary information (SI). Raw datasets can be obtained from the corresponding author upon a reasonable request. Supplementary information is available. See DOI: https://doi.org/10.1039/d6ra08323d.
