# Supplementary material for: A label-free fluorescent sensor for miR-21 based on sequentially coupled CHA–HCR dual-stage amplification and synergistic signal regulation by graphene oxide and SYBR Green I
Source: RSC Adv. 2026 Oct 19;16(48):64187–93. doi: 10.1039/d6ra08323d (PMC13678498; doi:10.1039/d6ra08323d)
Supplement: Supplementary file 1 [file RA-016-D6RA08323D-s001.pdf]

Supplementary Information (SI) for RSC Advances.
This journal is © The Royal Society of Chemistry

Supporting Information

A Label-Free Fluorescent Sensor for miR-21 Based on Sequentially Coupled CHA–HCR Dual-Stage Amplification and Synergistic Signal Regulation by Graphene Oxide and SYBR Green I

Supporting Tables

Table S1. The oligonucleotides used in this work

Name	Sequence (5'→3')
H1	CAGACTGATGTTGAGGGGCGACTTGAAATCAACATCAGTCTGATAAGCTA
H2	GGGGCGACTTGAAACATCAGACTGATGTTGATTTCAAGTCGCCCCTCAACATC
H3	GTTTCAAGTCGCCCCGAAGGAGGGGCGACT
H4	GGGGCGACTTGAAACAGTCGCCCCTCCTTC
miR-21	UAGCUUAUCAGACUGAUGUUGA
SM-21	UAGCUCAUCAGACUGAUGUUGA
DM-21	UAGCUCAUCAGACCGAUGUUGA
TM-21	UAGCUCAUCAGACCGAUGUUAA
miR-133a	AGCUGGUAAAATGGAACCAAAU
miR-128	UCACAGUGAACCGGUCUCUUUC
let-7a	UGAGGUAUAGGUUGUAUAGUU
miR-141	UAACACUGUCUGGUAAAGAUGG

Table S2. Algorithmic description of the detection workflow

Algorithmic description of the detection workflow
**Input:** miR-21
**Output:** fluorescence
**For** i = 1,..., int(miR-21) **do**
H1,H2,H3,H4/GO ← Adsorption (H1,H2,H3,H4, GO)
**While**(H1>0 and H2>0)
H1/H2,miR-21← Hybridization(miR-21,H1,H2)
dsDNA← Hybridization (H1-H2, H3,H4)
SG(i)← Signal (dsDNA)
**End while**
**End for**
sSG←sum(SG)
SG0←Leakage(miR-21)+Leakage(H1)+Leakage(H2)+Leakage(H3)+Leakage(H4)
**If (**sSG > SG0)
fluorescence ← true
**Else**
fluorescence ← false
**End if**
**End**

Table S3. Quantitative analysis of the background contribution of the CHA–HCR cascade

System	Replicate 1	Replicate 2	Replicate 3	Mean ± SD (a.u.)
With target CHA–HCR	26273	25307	24845	25475.0 ± 728.7
No target CHA–HCR	8117	8502	8224	8281.0 ± 198.7
Basal background	362	345	361	356.0 ± 9.5

To further quantify the residual background of the cascade system, the relative background contribution was calculated using the following equation:

$$\text{Relative background contribution (\%)} = \frac{F_{blank} - F_0}{F_{target} - F_0} \times 100$$

where F_{blank}, F_{target}, and F_0 are the mean fluorescence intensities of the no-target CHA–HCR system, the target-activated CHA–HCR system, and the basal background, respectively. Using the mean values in Table S3, the relative background contribution was 31.5%, and the background-corrected target-to-background fluorescence ratio was 3.17. The relative background contribution represents the overall residual background of the no-target system rather than intrinsic CHA–HCR leakage alone, because residual SGI binding to the hairpin structures and incomplete GO-mediated quenching also contribute to the measured background.

Table S4. Factors and levels

Level	HCR time (A)	H1/H2 concentration ratio (B)	Graphene oxide (GO) concentration (C)	SYBR Green I concentration (D)
1	120 min	0.75:1	10 μg/mL	1×
2	180 min	1:1	20 μg/mL	2×
3	240 min	1.25:1	30 μg/mL	3×

Table S5. Orthogonal Results

	HCR time (A)	H1/H2 concentration ratio (B)	GO concentration (C)	SYBR Green I concentration (D)	F/F_0
1	A_1	B_1	C_1	D_1	1.87
2	A_1	B_2	C_3	D_2	1.83
3	A_1	B_3	C_2	D_3	1.48
4	A_2	B_1	C_3	D_3	1.52
5	A_2	B_2	C_2	D_1	1.34
6	A_2	B_3	C_1	D_2	1.67
7	A_3	B_1	C_2	D_2	1.45
8	A_3	B_2	C_1	D_3	1.38
9	A_3	B_3	C_3	D_1	1.77
K1	5.18	4.85	4.92	4.98	
K2	4.53	4.55	4.28	4.96	
K3	4.61	4.92	5.12	4.38	
k1	1.73	1.62	1.64	1.66	
k2	1.51	1.52	1.43	1.65	
k3	1.54	1.64	1.70	1.46	
R	0.22	0.12	0.28	0.20	

Table S6. Comparison of the proposed method with other miR-21 detection methods

Method	Enzyme	Probe labeling	Cascade amplification	LOD	Ref.
nanoprobe	No	Yes	No	220 pM	1
APE1/CHA	Yes	Yes	No	250 pM	2
NIR-TMSDR	No	Yes	No	280 pM	3
CHA	No	Yes	No	330 pM	4
CHA-HCR	No	No	Yes	184 pM	This work

Supporting Figures

I. Computational Simulation Validation of the DNA Cascade Reaction

1. NUPACK Simulation Analysis

Before the wet-lab experiments, NUPACK was used to theoretically simulate the secondary structures of the hairpin probes and the hybridization behavior of the reaction system (Figure S1). Figure S1A–D shows the minimum free-energy (MFE) structures of the H1–H4 monomers at 37 °C. All probes adopted the expected closed hairpin conformations with high base-pairing probabilities. Their free energies ranged from -10.99 to -18.11 kcal/mol, indicating favorable thermodynamic stability and an ability to maintain the closed state effectively in the absence of the target. Figure S1E shows the MFE structure of the largest multistrand complex in the cascade reaction, which contained six strands. Its very high base-pairing probability and strongly negative free energy of -145.39 kcal/mol confirmed the high thermodynamic stability of the multistrand cascade product. Figure S1F further shows the equilibrium concentration distribution of different complexes. In the presence of miR-21, the reaction equilibrium shifted markedly toward the formation of long multistrand products. These theoretical simulation results validated the feasibility of the sequentially coupled CHA–HCR reaction pathway at the thermodynamic and molecular-structure levels and provided a theoretical basis for subsequent experimental validation.

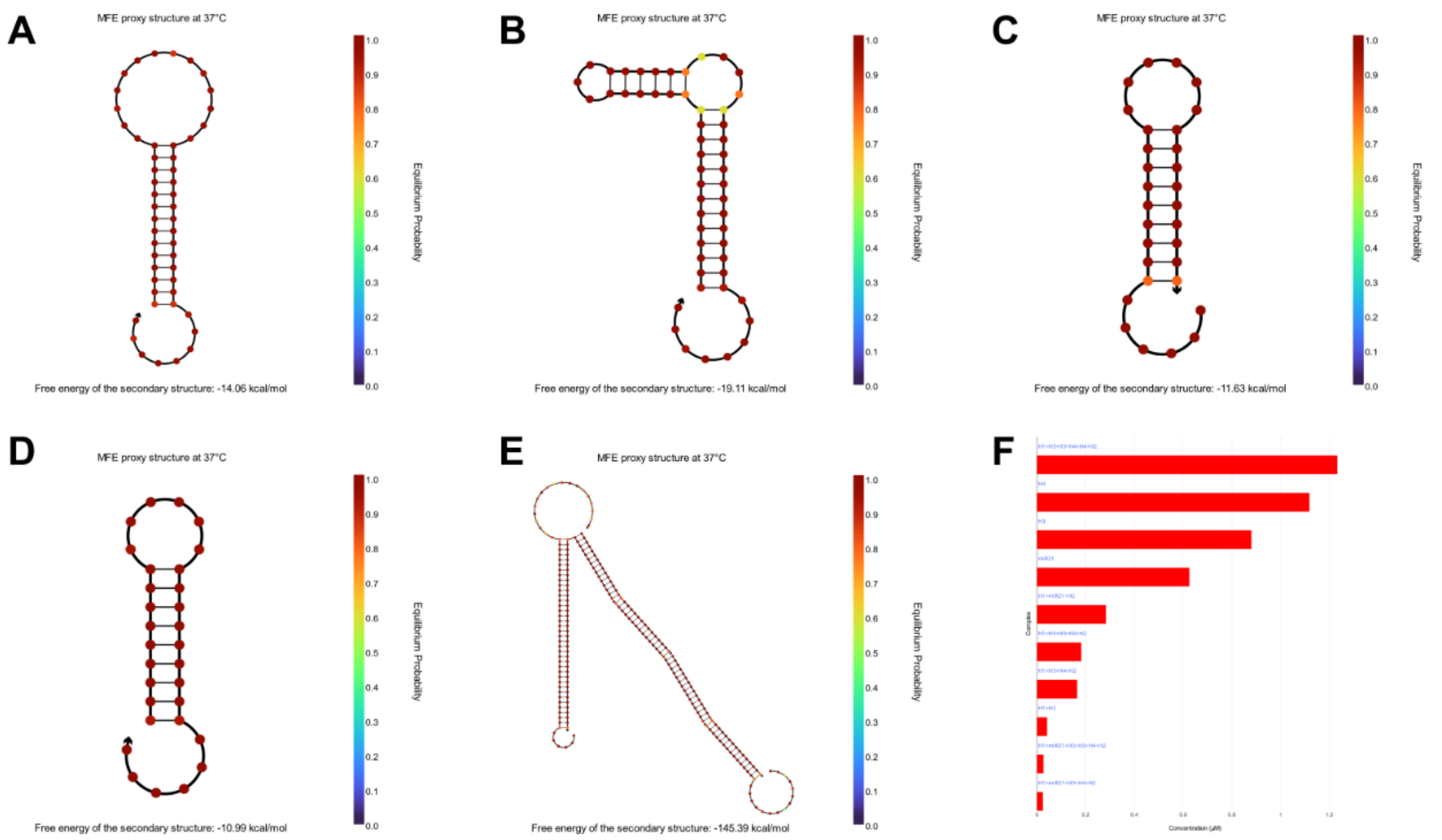

Figure S1. Secondary structures and standard free energies of the components involved in the strand-displacement reaction predicted by NUPACK. (A) Simulated MFE structure of H1. (B) Simulated MFE structure of H2. (C) Simulated MFE structure of H3. (D) Simulated MFE structure of H4. (E) Simulated MFE structure of the largest complex formed by the hairpin probes and the CHA–HCR output, containing six strands. (F) Equilibrium concentrations of different complexes in the test tube in the presence of miR-21 and the hairpin probes.

2. Kinetic Behavior Simulated Using Visual DSD

Visual DSD is a simulation platform for DNA strand-displacement-based molecular circuits that enables the modeling and kinetic analysis of programmable DNA circuits. Therefore, Visual DSD was used to simulate

the strand-displacement kinetics of CHA, HCR, and the sequentially coupled CHA–HCR reaction. The binding and unbinding rate constants used in the simulations were set to k = 0.0005 $nM^{-1}\ s^{-1}$ and u = 0.01 s^{-1}, respectively. Here, k describes the bimolecular binding of complementary domains, whereas u describes the dissociation of the corresponding bound domains. The bold black boxes represent input strands, and sp denotes an intermediate product generated during the reaction. In the CHA module (Figure S2A), the target first reacted with H1 to form intermediate sp1. After H2 was introduced, CHA product sp3 was generated through a strand-displacement reaction, accompanied by target release. Similarly, the CHA product served as the input strand for the downstream HCR (Figure S2B). After H3 and H4 were introduced, the progressively extended dsDNA products sp4, sp7, and sp8 were generated. Simulation of the complete cascade model (Figure S2C) showed that, in the presence of the target, the input strand was extended into the long-chain product H1–H2–(H3–H4)n during polymerization. This result confirmed the sequential triggering of downstream HCR by the upstream CHA product and demonstrated continuous signal transfer from target recycling to DNA polymerization and extension in
the cascade circuit.

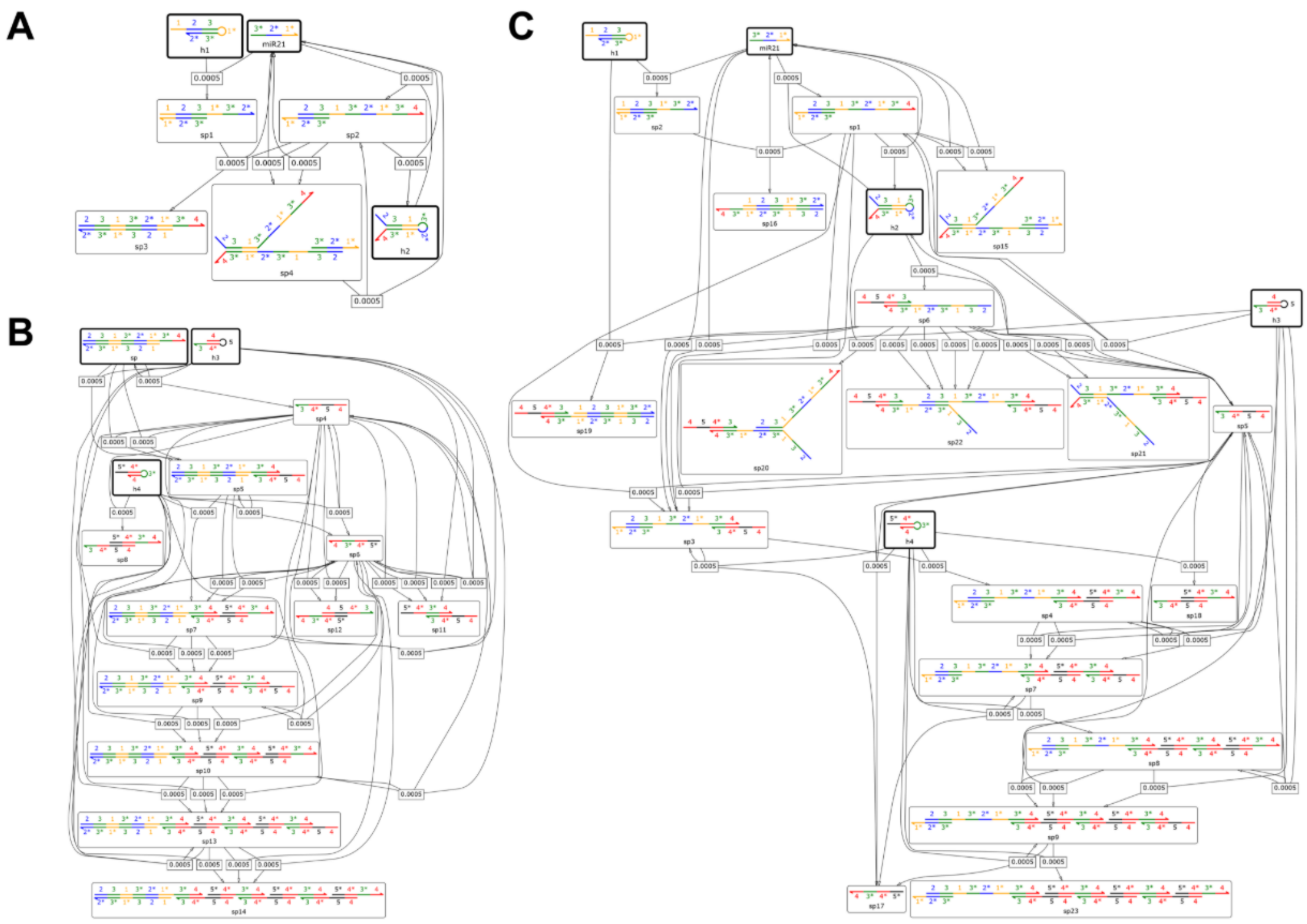

Figure S2. Reaction-network diagrams simulated using Visual DSD. (A) Simulated CHA reaction network. (B) Simulated HCR reaction network. (C) Simulated sequentially coupled CHA–HCR cascade reaction network.

II. Kinetic Characteristics of Fluorescence Quenching in the PGO–CHA–HCR System

To further investigate the interactions of graphene oxide (GO) with H1, H2, H3, and H4, time-dependent fluorescence-quenching experiments were performed in the presence of GO using H1, H2, H3, and H4 solutions without miR-21 (blank group) or with miR-21. As shown in Figure S3, when GO (25 μg/mL) was added to the blank solution, the fluorescence intensity decreased sharply at the beginning and was effectively quenched after 5 min. In contrast, after GO was added, the fluorescence intensity of

the H1, H2, H3, and H4 solution preincubated with the target at room temperature decreased more slowly than that of the blank group, indicating a markedly weaker interaction between the nicked dsDNA complexes and GO. At 20 min after GO addition, the fluorescence intensities of both the blank and experimental groups remained stable for approximately 1 h. Based on these observations, a 20-min incubation of GO with the CHA–HCR system was selected for subsequent analytical applications.

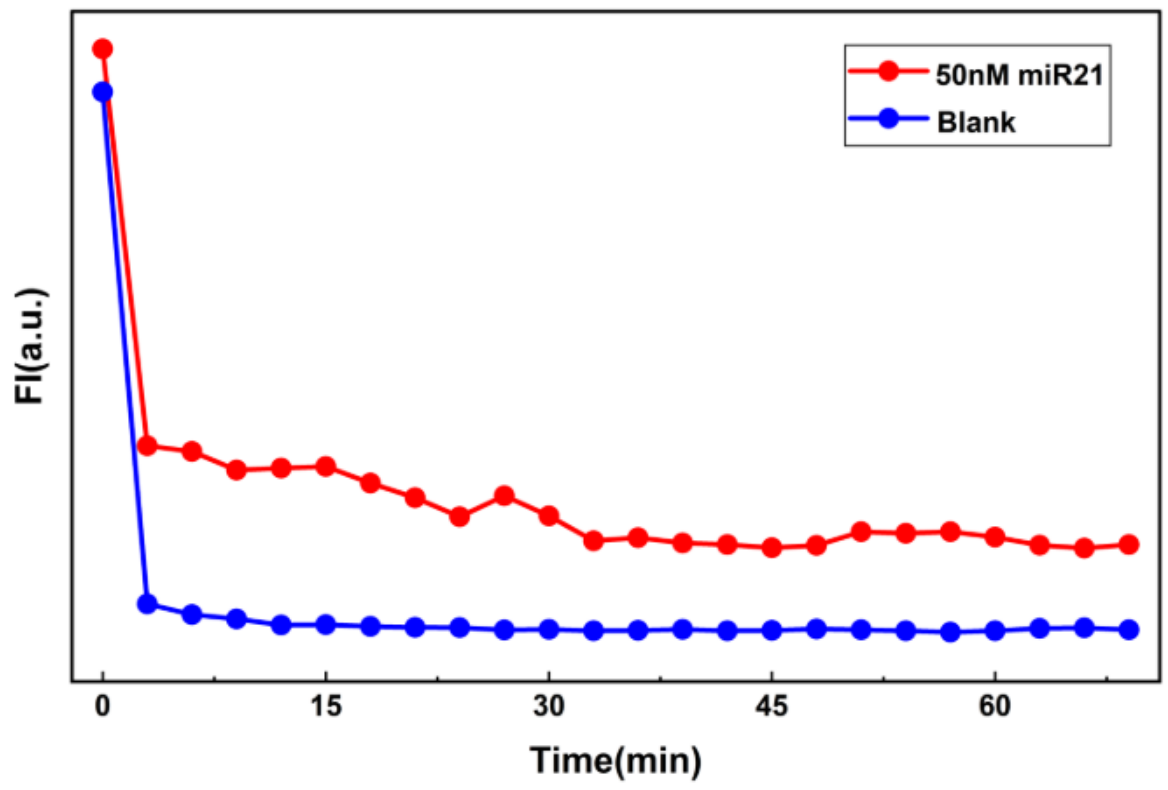

Figure S3. Time-dependent fluorescence quenching of H1, H2, H3, and H4 by graphene oxide after incubation with the target.

Ⅲ. Electrophoresis: unprocessed original gel images for Figure 2

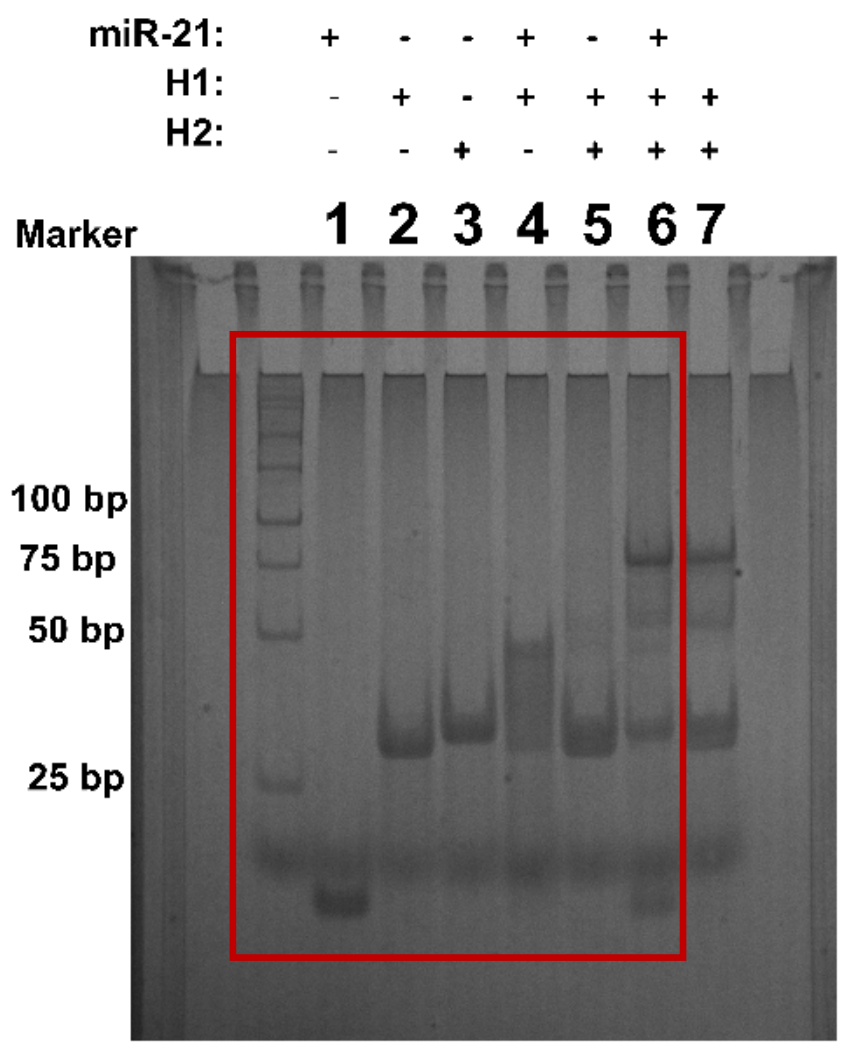

Figure S4. Uncropped raw gel image corresponding to Figure 2A in the main text.

Lane 1, miR-21; lane 2, H1; lane 3, H2; lane 4, miR-21 + H1; lane 5, H1 + H2; lane 6, miR-21 + H1 + H2; lane 7, annealed H1 + H2.

The region adopted in the main text is delineated by the red rectangle. One extra lane showing annealed H1/H2 complexes for validating CHA duplex formation was cropped out from Figure 2A. No lanes were rearranged.

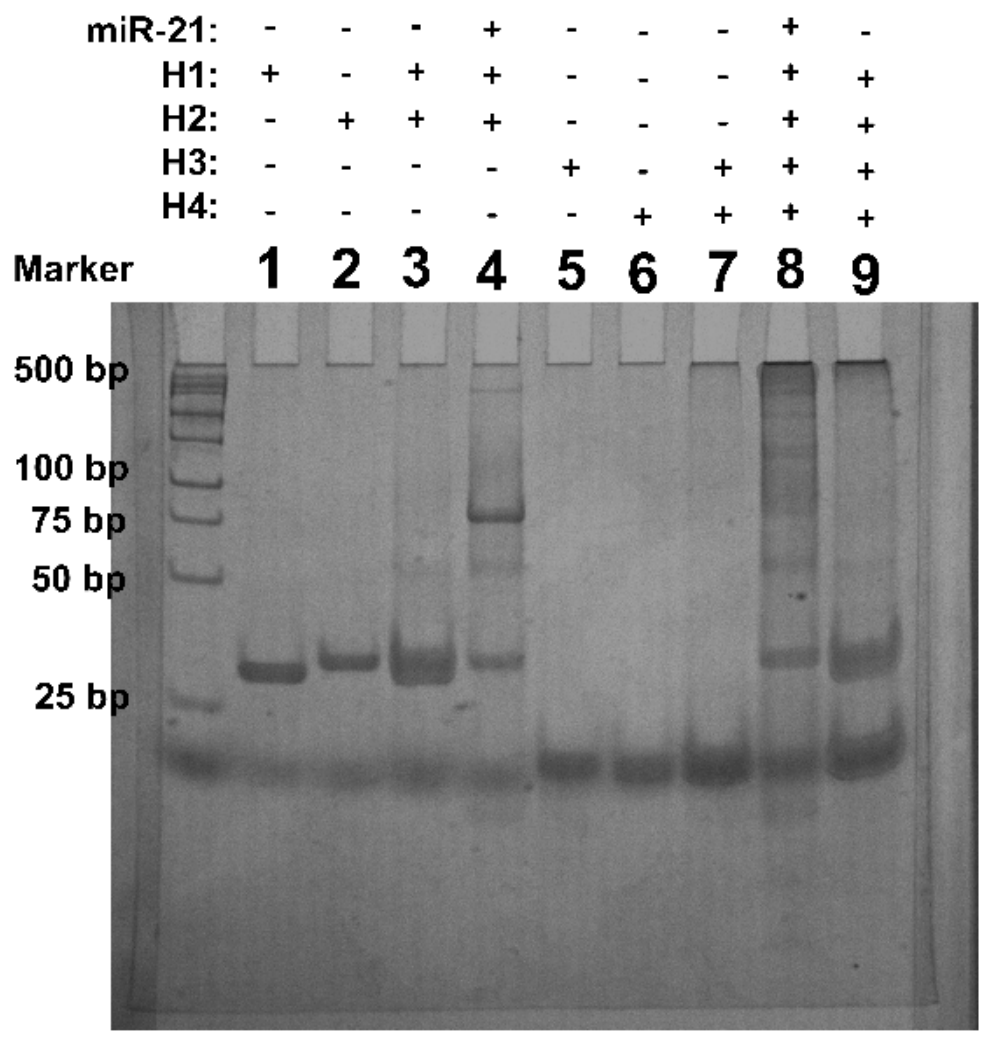

Figure S5. Uncropped raw gel image corresponding to Figure 2B in the main text.
Lane 1, H1; lane 2, H2; lane 3, H1 + H2; lane 4, miR-21 + H1 + H2; lane 5, H3; lane 6, H4; lane 7, H3 + H4; lane 8, miR-21 + H1 + H2 + H3 + H4; lane 9, H1 + H2 + H3 + H4.
No lanes were cropped or rearranged.

References

1. S. Q. Chai, W. Y. Lv, J. H. He, C. H. Li, Y. F. Li, C. M. Li and C. Z. Huang, *Anal. Chem.*, 2019, **91**, 6761-6768.
2. X. Lu, D. Li, Z. Luo and Y. Duan, *Analyst*, 2022, **147**, 2834-2842.
3. X. He, M. Yang, X. Zhang, B. Qiu and H. Jiang, *RSC Adv.*, 2026, **16**, 6844-6855.
4. W. Ouyang, Z. Liu, G. Zhang, Z. Chen, L. Guo, Z. Lin, B. Qiu and G. Chen, *Anal. Methods*, 2016, **8**, 8492-8497.
